# Saccharide Alterations in Spruce Wood Due to Thermal and Accelerated Aging Processes

**DOI:** 10.3390/polym17091265

**Published:** 2025-05-06

**Authors:** František Kačík, Tereza Jurczyková, Magdaléna Bálintová, Elena Kmeťová, Eva Výbohová, Danica Kačíková

**Affiliations:** 1 Department of Chemistry and Chemical Technology, Faculty of Wood Sciences and Technology, Technical University in Zvolen, 96001 Zvolen, Slovakia; kacik@is.tuzvo.sk (F.K.); vybohova@is.tuzvo.sk (E.V.); 2 Department of Wood Processing and Biomaterials, Faculty of Forestry and Wood Sciences, Czech University of Life Sciences Prague, Kamýcká 129, 16000 Prague, Czech Republic; 3 Institute for Sustainable and Circular Construction, Faculty of Civil Engineering, Technical University of Košice, Vysokoškolská 4, 04200 Košice, Slovakia; magdalena.balintova@tuke.sk; 4 Department of Fire Protection, Faculty of Wood Sciences and Technology, Technical University in Zvolen, 96001 Zvolen, Slovakia; xkmetovae@is.tuzvo.sk (E.K.); kacikova@is.tuzvo.sk (D.K.)

**Keywords:** cellulose, hemicelluloses, size exclusion chromatography, molecular weight distribution, crystallinity index, infrared spectroscopy

## Abstract

This work is devoted to the changes in polysaccharides in thermally treated wood after its accelerated aging with the aim of its optimal utilization after its original use has ended. Spruce wood samples were treated by the Thermowood process at temperatures of 160 °C, 180 °C, and 210 °C and subjected to accelerated aging in wet mode. The influence of treatment temperature and accelerated aging was monitored by wet chemistry, high-performance liquid chromatography (HPLC), X-ray diffraction (XRD), size exclusion chromatography (SEC), and Fourier-transform infrared spectroscopy (FTIR). During thermal treatment, hemicelluloses are mainly degraded. At the temperature of 210 °C, aromatic compounds formed as degradation products of lignin and hemicelluloses bind to cellulose fibers and increase cellulose yield. Preferential decomposition of the amorphous portion of cellulose leads to an increase in its crystallinity, while higher temperatures cause degradation of the crystal lattice. The degree of polymerization in both cellulose and hemicelluloses decreases due to the cleavage of glycosidic bonds. Accelerated aging does not significantly affect the changes in polysaccharides. The results obtained can be used in the processing of cellulose and hemicelluloses from thermally modified wood at the end of its life cycle in various industrial fields.

## 1. Introduction

Thermal modification, which exhibits low greenhouse gas emissions and energy consumption compared to traditional chemical treatments [1], is integral to modern wood preservation and enhancement strategies [2]. The process involves elevating wood temperatures (ranging from 160 °C to 260 °C) in a controlled environment [3], primarily altering its polysaccharide composition, specifically hemicelluloses and cellulose. These changes significantly influence, e.g., hygroscopicity and dimensional stability [4,5,6], resistance to biological degradation [7,8,9], mechanical properties [10,11,12,13], density, color, odor, gluability, and coating performance [14,15] but also provide pathways for its sustainable utilization at the end of its life cycle, mainly due to its altered chemical and physical properties.

Cellulose, as a highly crystalline homopolymer of β-(1–4)-linked D-glucose units, provides tensile as well as compressive strength and rigidity. At the same time, hemicelluloses, as shorter and branched heteroglycans of several different neutral and acidic monosaccharides, offer flexibility and can mitigate brittleness due to their amorphous structure [16,17]. Their interactions with cellulose and lignin are essential for maintaining wood’s mechanical properties and structural cohesion [18]. Hemicelluloses are more prone to thermal degradation than cellulose and lignin, which impacts the chemical composition of wood during natural aging and heat treatment processes [19]. Under temperatures up to 190 °C, hemicelluloses undergo depolymerization and deacetylation. The amount of hydroxyl groups available in wood decreases, which stabilizes wood against moisture fluctuations but also reduces its hygroscopic capabilities [20,21,22]. Cellulose crystallinity (TCI) remains stable up to 210 °C or 220 °C, and then increases, likely due to degradation of hemicelluloses and amorphous cellulose [23,24] and moreover due to loss of mass [25]. Since the increased crystallinity theoretically contributes significantly to wood strength, shortening of cellulose chains through cleavage of glycosidic bonds, resulting in a lower degree of polymerization (DP), which occurs already up to 120 °C [26], leads to a decrease in the mechanical performance and structural integrity of thermally modified wood [22,27]. Earlier findings [28] also confirm the formation of volatile compounds during the degradation of both polysaccharides. The stability and degradation behavior of these saccharides play a key role in determining the performance and long-term durability of thermally modified wood.

Upon reaching the end of its useful life, thermally treated wood can potentially be recycled into new materials, aligning with the principles of a circular economy [29,30]. For instance, it can serve as a reinforcing agent or bioadditive in various applications [31]. In the paper and textile industries, wood fibers derived from thermally treated wood are particularly valuable due to their enhanced properties [32,33]. Moreover, these materials can be utilized as fillers in composite wood panels, hydrogels, and flexible packaging films for cosmetics, pharmaceuticals, and food applications [34,35]. Residual cellulose-rich fractions from thermally treated wood can be hydrolyzed into sugars and subsequently fermented into bioethanol or other biofuels, thus promoting renewable energy solutions [36,37]. The lower moisture content and higher energy density of thermally treated wood render it suitable for energy recovery through combustion or gasification [38]. Additionally, pyrolyzed thermally treated wood can be converted into biochar, which enhances soil water retention and nutrient retention, particularly in nutrient-poor or dry areas [39]. Biochar not only contributes to agricultural benefits but also serves as a precursor for producing activated carbon, which is significant in air and water purification systems [40]. Furthermore, the degraded polysaccharides remaining after thermal treatment can act as feedstock for the chemical industry, facilitating the green synthesis of various chemicals [41,42]. This multifaceted potential of thermally treated wood not only underscores its versatility as a resource but also highlights its significance in promoting sustainability across several sectors.

To establish a robust foundation for the further processing of aged thermally modified wood, specifically regarding the reuse of modified wood waste, it is imperative to gain precise insights into the composition of the raw material. This necessitates an extensive investigation into the influence of accelerated aging on wood that has already undergone thermal treatment. Particular attention should be given to the combined effects of temperature and UV/rain exposure on the structural integrity and quantitative and qualitative analysis of saccharides present in the wood matrix.

The primary objective of this research is to evaluate how thermal treatment at various temperatures, along with subsequent accelerated aging simulations, affects the chemical composition and structural characteristics of saccharides in spruce wood (Picea abies). The study will place significant emphasis on utilizing various instrumental methods, which underscore the advantages of employing a multi-faceted analytical approach. Techniques such as Fourier-transform infrared spectroscopy (FTIR), X-ray diffraction (XRD), size exclusion chromatography (SEC), and high-performance liquid chromatography (HPLC) will be integrated. This combination of methodologies will provide comprehensive insights into both the chemical alterations and structural transformations experienced by the wood during thermal modification and subsequent aging processes.

By systematically investigating these factors, this study aims to enhance the understanding of the behavior of thermally modified wood under aging conditions, thereby contributing to improved design methodologies for treatments tailored to specific end-uses of lignocellulosic biomass. Ultimately, the findings will inform better strategies for the sustainable reuse of thermally modified wood, facilitating its application in various industries while promoting environmental sustainability and resource efficiency.

## 2. Materials and Methods

### 2.1. Material

Four sets of specimens (each 10 pieces) were prepared from spruce wood (*Picea abies*, Karst.) of dimensions 200 mm × 100 mm × 20 mm (length × width × thickness). One set consisted of samples without thermal treatment (reference, REF). The other three sets were thermally treated at 160, 180, and 210 °C (160-TW, 180-TW, and 210-TW). All samples were conditioned at 65% relative humidity and 20 °C before accelerated aging. The samples were grouped in a way that considered the heterogeneity of spruce wood. The number was chosen according to the capacity of the equipment for thermal treatment and accelerated aging.

### 2.2. Accelerated Aging

Accelerated wood aging of thermally treated wood was conducted in a Q-SUN Xe-3-HS xenon test chamber (Q-Lab Europe, Ltd., Bolton, UK). The test material placed in the xenon test chamber was regularly rotated according to the recommended schedule to ensure equal radiation intensity and heat for all specimens. The aging conditions in the xenon chamber were according to ASTM G 155 [43]. The outdoor (wet) mode was used to simulate the conditions in which wood is exposed to radiation and rain (Table 1).

Samples were denoted as 160-TW-XE, 180-TW-XE, and 210-TW-XE.

### 2.3. Chemical Analyses

Samples were ground to a particle size of 200–300 µm using a POLYMIX PX-MFC 90D laboratory mill (Kinematica, Luzern, Switzerland) and extracted with a mixture of absolute ethanol (Merck, Darmstadt, Germany) and toluene (Merck, Germany) (1:0.427, *v*/*v*) (ASTM D1107-21 [44]). Cellulose was determined according to Seifert [45] and holocellulose according to Wise et al. [46]. Hemicelluloses were calculated as the difference between the holocellulose and cellulose contents. Measurements were made in four replicates per sample. The results are expressed as oven-dry mass per unextracted wood.

Structural carbohydrates were determined by high-performance liquid chromatography (HPLC) using a Biorad Aminex HPX-87P (Bio-Rad Laboratories, Hercules, CA, USA) column according to Sluiter et al. [47].

### 2.4. XRD Analysis

The crystallinity index (CI) of the samples was determined by the X-ray diffraction (XRD) technique using a Bruker D2 Phaser X-ray powder diffractometer (Bruker AXS, GmbH, Karlsruhe, Germany). The diffraction patterns were recorded using CuKα radiation (λ = 0.154060 nm), a voltage of 30 kV, and a current of 10 mA. The equipment was operated in continuous scan mode with a step size of 0.025° (2Θ), a step time of 5 s, and a scan range 5° < 2Θ < 40°. The CI was calculated by the amorphous subtraction method using the Bruker DIFFRAC.EVA version 5.2 software [48].

### 2.5. Size Exclusion Chromatography

Molecular weights and molecular weight distribution (MWD) of cellulose were analyzed using a modified method [49]. Briefly, isolated cellulose samples (20 mg) were derivatized with phenyl isocyanate (1 mL phenyl isocyanate and 6 mL pyridine) in a sealed dropping flask to obtain cellulose tricarbanilates (CTC) at 80 °C for 48 h. After cooling to laboratory temperature, 2 mL of methanol was added to dissolve the excess phenyl isocyanate. According to other authors, the derivatization conditions used in this work do not affect the molecular weight of cellulose [50,51]. Samples were filtered with a glass filter (0.7 µm) and size exclusion chromatography (SEC) analyses were performed at 35 °C with tetrahydrofuran (mobile phase) at a flow rate of 1 mL·min^−1^ on two PLgel, 10 μm, 7.5 × 300 mm, MIXED B columns, in combination with a PLgel, 10 μm, 7.5 × 50 mm, GUARD column (Agilent, Santa Clara, CA, USA). Two CTC derivatives were prepared for each sample, and each derivative was chromatographed twice.

SEC analysis of hemicelluloses was performed on TSKgel SuperMultiporePW-N HPLC column (4 µm, 6 × 150 mm) (Tosoh Bioscience, Griesheim, Germany) in a mobile phase of 0.02 M sodium hydroxide/0.2 M sodium acetate solution (at pH 11.8). The system was calibrated with Polysaccharide Calibration Kit (Agilent, Santa Clara, CA, USA), consisting of oligosaccharides and pullulans. Hemicelluloses were extracted using a modified method [52]. Briefly, 100 mg of holocellulose was inserted into a 2 mL syringe filled with glass wool at the needle end, and 1 mL of 17.5% aqueous sodium hydroxide was added. Extraction was carried out for 3 h at an ambient temperature. The extract was then rapidly filtered (PTFE filter, 0.45 µm) and immediately injected into the Agilent 1200 HPLC chromatograph (Agilent Technologies, Santa Clara, CA, USA).

### 2.6. ATR-FTIR Analysis

Fourier-transform infrared spectroscopy (FTIR) of isolated cellulose was performed on a Nicolet iS10 FT-IR spectrometer (Thermo Fisher Scientific Inc., Waltham, MA, USA) with the Smart iTR ATR accessory. Spectra were collected in the absorption mode between 4000 and 650 cm^−1^ by accumulating 32 scans with a resolution of 4 cm^−1^ using a diamond crystal. All analyses were carried out in four replicates.

## 3. Results and Discussion

Wet chemistry analyses of untreated and modified spruce wood show that thermal modification reduces the polysaccharides content, in particular by the decomposition of hemicelluloses (Table 2). Hemicelluloses content drops by 75.59% at 210 °C in thermally treated specimens and by 80.48% in thermally treated and aged specimens. A temperature of 160 °C has only a small effect on changes in hemicelluloses; more significant changes occur at a temperature of 180 °C, and especially at a temperature of 210 °C. Accelerated aging due to UV radiation and water exposure affects the degradation of hemicelluloses and their leaching from the wood. The presented results are in line with published data indicating a decrease in spruce wood polysaccharides during thermal treatment, especially hemicelluloses [28]. Moreover, the thermal treatment causes shortening of the polysaccharide fibers and reduced width, which was even more clearly manifested during accelerated aging [53].

The cellulose content increases at 210 °C, possibly due to aggregation with lignin and degradation products of hemicelluloses [54]. This phenomenon is supported by the results of carbohydrate analysis, where the amount of glucose decreases with increasing thermal treatment temperature (Table 3). In addition, typical bands for aromatic compounds (1604, 1512, 1261 cm^−1^) appear in the FTIR spectra of cellulose at 210 °C. Our results are consistent with the changes observed in hygrothermally modified holocellulose—decreased monosaccharides content and the appearance of signals in FTIR spectra indicating the formation of the aromatic compounds [55].

Mannose and galactose are found in greater amounts than in deciduous wood in the hemicelluloses of coniferous wood. The ratio of non-glucose carbohydrates mannose:xylose:galactose:arabinose is 13.6:5.6:2.8:1.2. In spruce wood, the predominant hemicelluloses are acetylated galactoglucomannans and arabinoglucuronoxylans, with minor hemicelluloses such as arabinogalactans [16,56]. In wood, carbohydrates are lost during thermal treatment, but their decomposition rate varies. The most stable is glucose, which is mainly found in cellulose (Table 2). In hemicelluloses it is found in galactoglucomannan, where the ratio mannose:glucose:galactose = 3.1:1:0.7 [56]. Galactose breaks down the fastest, while mannose is the most stable (Table 4).

**Figure 1:**
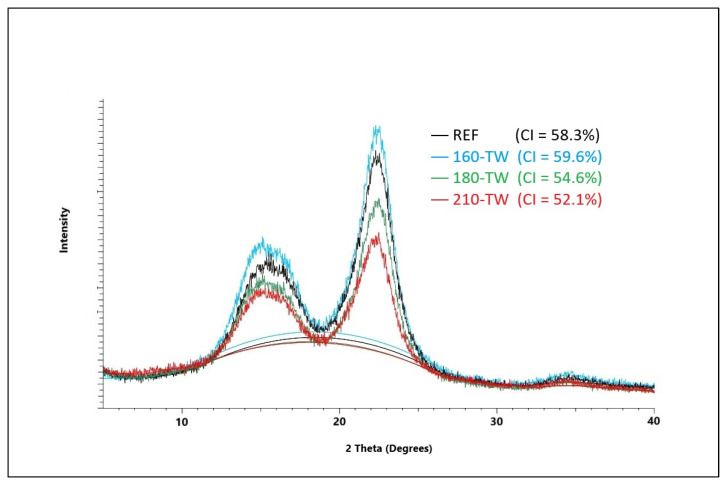
XRD diffractogram of spruce wood cellulose before and after thermal treatment.

**Figure 2:**
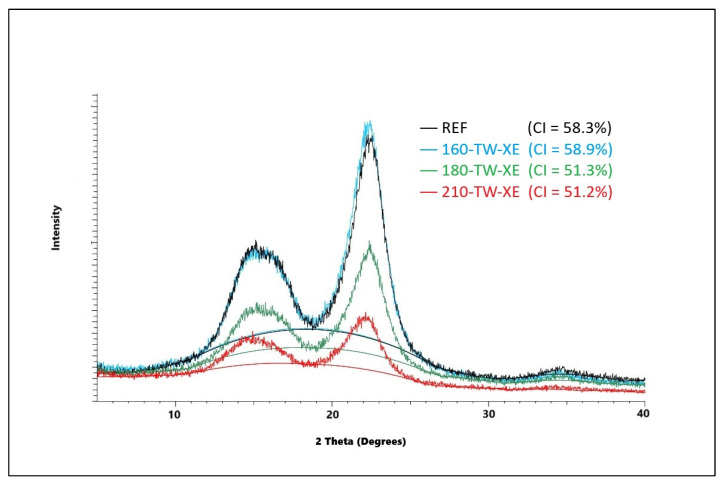
XRD diffractogram of spruce wood cellulose before and after thermal treatment and accelerated aging.

The crystallinity of cellulose significantly affects its mechanical and chemical properties. During thermal treatment, the crystallinity of cellulose changes, and depending on the treatment conditions, the crystallinity increases, but it can also decrease. The amorphous part of cellulose is more sensitive to degradation at higher temperatures, which leads to a relative increase in the crystalline fraction [57]. During mild torrefaction, a slight decrease in cellulose crystallinity was observed, attributed to its amorphization on crystallite surfaces because of acid hydrolysis and free radical reactions resulting in the homolytic splitting of glycosidic bonds [58]. In our experiments, crystallinity first increases due to faster degradation of the amorphous part of cellulose; at temperatures of 180 °C, the cellulose crystal lattice degrades, and its crystallinity decreases; its values are lower in aged wood (Figure 1 and Figure 2). These results are coherent with previously reported results for spruce wood thermally modified at a similar range of temperatures [59]. Lower crystallinity of cellulose after accelerated aging provides better conditions for chemical reactions and thus for the preparation of various cellulose derivatives. It also affects photo-degradation, thermal stability, hygroscopicity, and hydrolysis of wood [60,61].

Size exclusion chromatography is a useful method for determining the molecular weight of cellulose and for monitoring its molecular weight distribution—MWD, or molar mass distribution—MMD. *M*_n_ is the number-averaged molecular weight, which emphasizes the low-molar mass material; *M*_w_ is the weight-averaged molecular weight, which reflects the high molar mass material; and *M*_z_ is sensitive to the highest molar mass fraction of a sample [50]. This allowed a better monitoring of the thermal-stress effects concerning the respective molar mass fractions [62].

**Figure 3:**
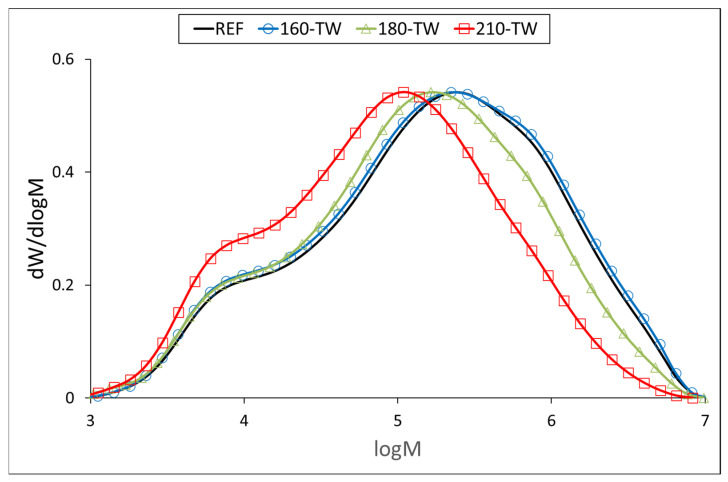
Molar mass distribution curves of cellulose tricarbanilates from thermally treated wood.

**Figure 4:**
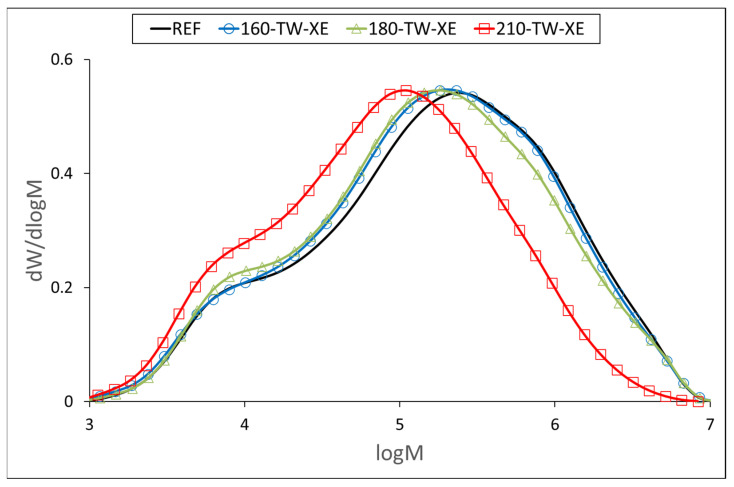
Molar mass distribution curves of cellulose tricarbanilates from thermally treated and aged wood.

At 160 °C, the degree of polymerization (DP) of cellulose increases slightly (Table 5, Figure 3 and Figure 4), which may be due to the aggregation of cellulose chains, a phenomenon also observed during kraft pulping and hydrothermal treatment [63,64]. Higher temperatures cause the disintegration of the aggregates and significant depolymerization of the cellulose chains, resulting from the cleavage of glycosidic bonds. Cellulose DP at 210 °C was reduced by half, while no significant difference was observed between the samples before and after accelerated aging (Table 5). Similar results were reported for mild torrefaction of eucalyptus wood, where DP values decreased from 1300 to 530 and from 1330 to 590, respectively [58].

Research on hemicelluloses is of great importance because of their potential applications in food, healthcare, paper, textile, and cosmetics industries, fuel additives, plastics, and chemical production [65,66,67]. In addition to the ratio of individual monosaccharides in hemicelluloses, their molecular weight also has a significant impact on their use. In addition to the type of biomass, the method of extraction also influences its value. The extraction of hemicelluloses has been studied by many techniques such as steam explosion, treatment with alkali or dilute acid, hot water extraction, and pressurized water extraction [56,68]. The average molecular weights isolated from spruce pulp were 39,000, 43,000, and 46,000 g·mol^−1^, respectively [69,70]; from spruce sapwood, the average of isolated hemicelluloses was within 20,000–70,000 g·mol^−1^ [71].

**Figure 5:**
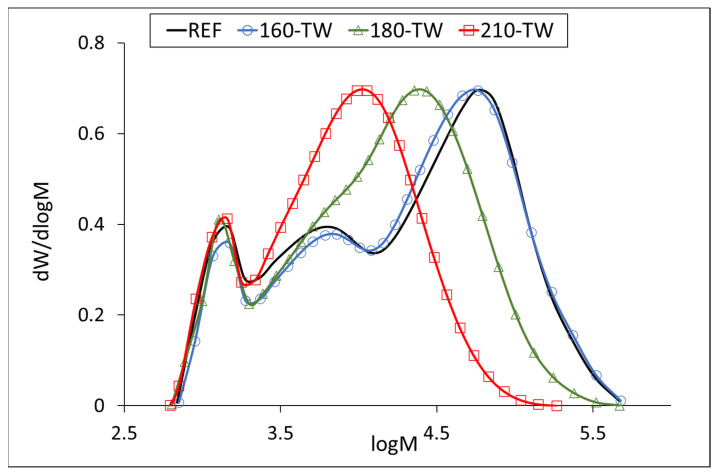
Molar mass distribution curves of hemicelluloses from thermally treated wood.

**Figure 6:**
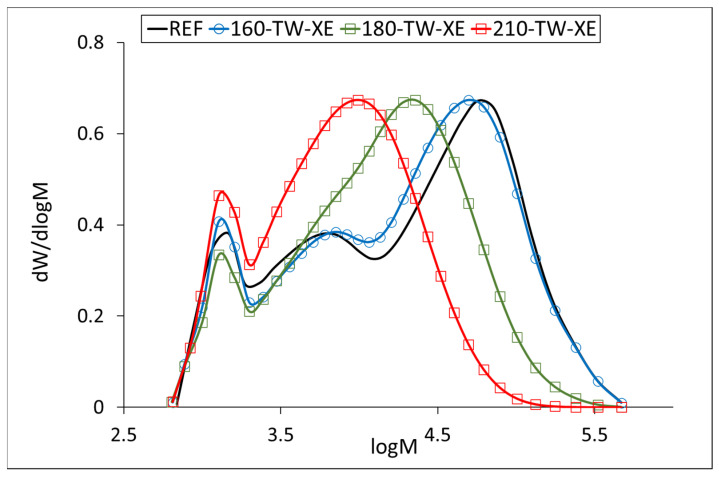
Molar mass distribution curves of hemicelluloses from thermally treated and aged wood.

Our results for the average molecular weight of the hemicelluloses isolated from the untreated sample (about 45,000 g·mol^−1^) agree with the published ones. The temperature of 160 °C degrades the hemicellulose chains only slightly, while accelerated aging has a more significant effect at this temperature. A similar phenomenon can be observed at 180 °C, where the decrease in molecular weight is more pronounced, 40% after thermal treatment and 45% after accelerated aging. At 210 °C, the decrease was similar for both samples, around 70% (Table 6). The molecular weight distribution curves of hemicelluloses from the untreated sample show three fractions with molecular weights of approximately 57,000 g·mol^−1^ (REF, 160-TW), 6000 g·mol^−1^ (REF, 160-TW), and 1300 g·mol^−1^ (all samples). The maximum of the fractions with the highest values decreases in the heat-treated samples from approximately 57,000 g·mol^−1^ (REF, 160-TW) to 22,700 g·mol^−1^ (180-TW) and to 11,300 g·mol^−1^ (210-TW). In aged samples, the decrease in molecular weight of these fractions is somewhat faster, from approximately 57,000 g·mol^−1^ (REF) to 49,800 g·mol^−1^ (160-TW-XE), 22,600 g·mol^−1^ (180-TW-XE), and 9800 g·mol^−1^ (210-TW-XE). The peak with the middle fractions shows a shoulder in the 180-TW sample, while in the 210-TW sample, it is overlapped by the peak with the highest molecular weight. The peak with the lowest molecular weight has the same value in both types of samples (around 1300 g·mol^−1^) (Figure 5 and Figure 6).

**Figure 7:**
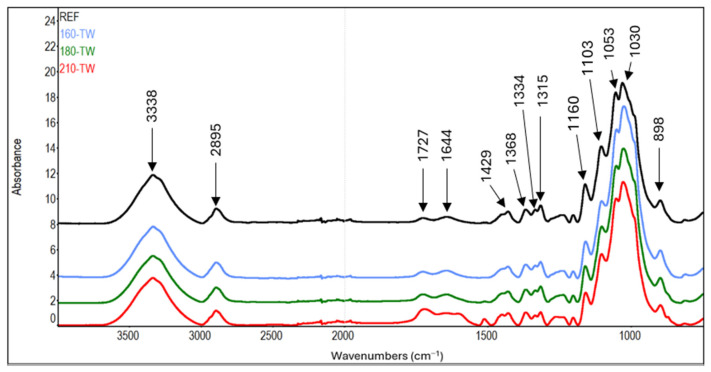
FTIR spectra of cellulose from thermally treated spruce wood.

**Figure 8:**
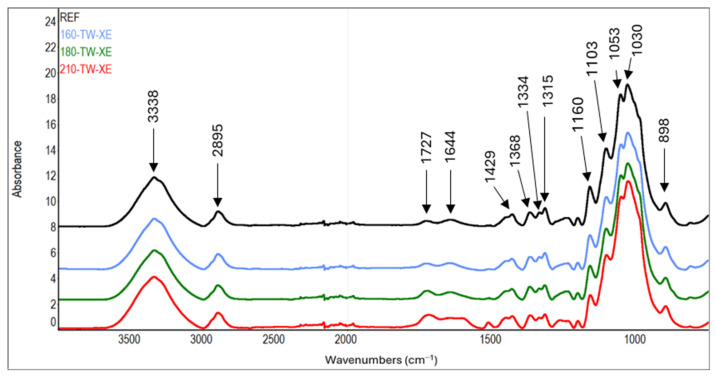
FTIR spectra of cellulose from thermally treated and aged spruce wood.

In the FTIR spectra of cellulose, the intensity growth of the absorption band of unconjugated carbonyl groups at a wavenumber of 1727 cm^−1^ can be observed with increasing treatment temperature (Table 7, Figure 7 and Figure 8). This finding is in accordance with the results of another study [72,73]. It may mean forming new carbonyl groups via oxidation reactions, which naturally occur in thermally modified wood [15,74]. In addition, slight degradation processes of cellulose can also be observed. These are manifested by a decrease in the intensity of C−O−C vibrations at 1160 cm^−1^, OH in-plane bending at 1334 cm^−1^, and CH_2_ wagging at 1315 cm^−1^. In contrast, during the natural aging of thermally treated pine wood, no significant differences were observed in this region of infrared spectra, which may also be due to a different method of cellulose isolation [75].

Another interesting finding is the occurrence of absorption bands belonging to aromatic skeletal vibrations (1512 cm^−1^) and C_aryl_-O bond (1261 cm^−1^) in the spectra of cellulose from samples modified at higher temperatures. The intensities of the mentioned bands reach their maximum in samples modified at 210 °C. In the case of aromatic skeletal vibrations, the intensity of the absorption band after thermal treatment increases eleven times, and in the case of C_aryl_-O bond, it increases by 68% compared to the reference sample. These findings indicate the formation of aromatic aggregates by the binding of lignin and hemicelluloses degradation products to cellulose fibers [55].

In follow-up research, we want to further expand the currently identified and experimentally confirmed information and trends and specifically explore the relationship between structural and molecular changes in polysaccharides and the mechanical properties of thermally treated and aged wood, such as bending strength, stiffness, or fracture resistance, in order better to understand the practical implications of the observed degradation. Furthermore, we plan to evaluate the utilization of degraded polysaccharides for targeted applications, including biopolymeric materials, substrates for enzymatic hydrolysis, or direct use in the textile and papermaking industry. Lastly, we also plan to assess the influence of additional related environmental factors, such as moisture and drying cycles or changing intensities of UV radiation, on the structure and reactivity of polysaccharides under real long-term service conditions.

## 4. Conclusions

In this work, the changes in polysaccharides of heat-treated spruce wood under the influence of different temperatures and accelerated aging were monitored. Temperature has a significant effect on the decrease in polysaccharides, especially hemicelluloses, the change in which is insignificant at a temperature of 160 °C, then their amount decreases sharply. More significant changes in cellulose occur only at temperatures above 200 °C. At a temperature of 210 °C, aromatic compounds bind to cellulose fibers, which are formed as degradation products of lignin and hemicelluloses. Of the non-glucose carbohydrates, galactose decomposes the fastest, and mannose is the most stable. The crystallinity of cellulose increases at a temperature of 160 °C as a result of the degradation of its amorphous part, and at higher temperatures, it decreases due to the degradation of the crystalline part of cellulose. Thermal modification leads to a significant decrease in the degree of polymerization of cellulose and hemicelluloses. Accelerated aging, in contrast to temperature, has no significant effect on the changes in polysaccharide content. The results obtained can be used in the processing of cellulose and hemicelluloses in various fields, e.g., pulp and paper production, pharmaceuticals, and plastics. In addition, lower cellulose crystallinity and lower molecular weights of both cellulose and hemicelluloses after accelerated aging provide the prerequisites for better hydrolysis (acidic and especially enzymatic) for the production of second-generation bioethanol. These observations create a prerequisite for future research that could address in more detail the physico-mechanical consequences of the detected chemical changes, which are already generally presented in the Introduction part. We also, at least briefly, mention possible practical applications of wood containing degraded polysaccharides, e.g., for the production of bioethanol or composites; thus, the study touches on broader aspects of the material’s usability.

## Figures and Tables

**Table 1:** The accelerated aging parameters according to the standard ASTM G 155 “wet mode”.

Step	Mode	Radiation Intensity (W·m^−2^)	Black Panel Temperature (°C)	Air Temperature (°C)	Relative Air Humidity (%)	Time (min)
1	Radiation	0.35	63	48	30	102
2	Radiation + water spraying	0.35	63	48	90	18

**Table 2:** Yields of holocellulose, cellulose, and hemicelluloses in the analyzed samples (% odw, SD are in parentheses).

*T* (°)	TW			TW-XE		
	Holo- Cellulose	Cellulose	Hemi- Celluloses	Holo- Cellulose	Cellulose	Hemi- Celluloses
REF	77.43 (0.61)	45.35 (0.26)	32.07 (0.68)	77.43 (0.61)	45.35 (0.26)	32.07 (0.68)
160	76.29 (0.78)	45.48 (0.14)	30.81 (0.81)	76.38 (0.36)	45.38 (0.12)	31.00 (0.40)
180	66.93 (0.61)	46.33 (0.21)	20.59 (0.81)	62.72 (0.36)	45.23 (0.37)	17.49 (0.54)
210	58.39 (0.12)	58.39 (0.17)	7.83 (0.16)	58.82 (0.22)	52.56 (0.11)	6.26 (0.12)

**Table 3:** Structural carbohydrates in spruce specimens (% in wood, SD are in parentheses).

Sample	Glucose (GLC)	Xylose (XYL)	Galactose (GAL)	Arabinose (ARA)	Mannose (MAN)	Total
REF	48.24 (0.83)	6.52 (0.10)	3.47 (0.03)	3.32 (0.31)	11.32 (0.16)	72.87 (0.99)
160-TW	44.31 (0.53)	4.98 (0.11)	3.09 (0.09)	1.61 (0.08)	10.48 (0.16)	64.47 (0.89)
180-TW	41.46 (0.06)	4.94 (0.06)	1.84 (0.08)	1.71 (0.06)	9.00 (0.13)	58.95 (0.17)
210-TW	40.38 (0.22)	3.57 (0.05)	1.00 (0.07)	1.11 (0.09)	7.82 (0.16)	53.87 (0.38)
160-TW-XE	43.07 (0.32)	4.92 (0.21)	2.91 (0.04)	1.64 (0.33)	10.40 (0.41)	62.94 (1.14)
180-TW-XE	38.18 (0.57)	5.18 (0.10)	2.63 (0.03)	1.55 (0.05)	7.81 (0.21)	55.35 (0.88)
210-TW-XE	37.44 (0.34)	3.78 (0.12)	1.42 (0.05)	0.88 (0.03)	7.12 (0.09)	50.64 (0.56)

**Table 4:** Structural carbohydrates in spruce specimens (% of total sugars, SD are in the parentheses).

Sample	Glucose (GLC)	Xylose (XYL)	Galactose (GAL)	Arabinose (ARA)	Mannose (MAN)
REF	64.75 (0.36)	9.33 (0.13)	4.96 (0.05)	4.76 (0.42)	16.21 (0.16)
160-TW	68.73 (0.16)	7.73 (0.06)	4.79 (0.14)	2.50 (0.09)	16.25 (0.04)
180-TW	70.33 (0.11)	8.39 (0.09)	3.12 (0.13)	2.89 (0.10)	15.27 (0.21)
210-TW	74.96 (0.45)	6.63 (0.07)	1.85 (0.12)	2.06 (0.16)	14.51 (0.24)
160-TW-XE	68.44 (0.99)	7.82 (0.23)	4.62 (0.03)	2.60 (0.47)	16.52 (0.35)
180-TW-XE	68.98 (0.18)	9.35 (0.05)	4.76 (0.13)	2.80 (0.05)	14.11 (0.19)
210-TW-XE	73.94 (0.24)	7.46 (0.16)	2.80 (0.08)	1.73 (0.07)	14.07 (0.03)

**Table 5:** SEC results of spruce wood cellulose (g·mol^−1^, SD are in the parentheses).

Sample	*M*_n_	*M*_w_	*M*_z_	PDI	DP
REF	13,590 (252)	196,859 (9276)	703,596 (12,596)	14.48 (0.42)	1215 (57)
160-TW	13,531 (138)	215,639 (3374)	750,340 (8230)	15.94 (0.39)	1338 (20)
180-TW	12,061 (286)	164,887 (5165)	629,976 (19,977)	13.67 (0.11)	1027 (32)
210-TW	8720 (109)	99,330 (2559)	431,606 (9563)	11.39 (0.15)	610 (18)
160-TW-XE	13,532 (607)	214,154 (20,431)	754,195 (42,805)	15.83 (0.80)	1322 (126)
180-TW-XE	13,072 (316)	191,052 (2551)	734,342 (4990)	14.62 (0.16)	1179 (16)
210-TW-XE	8769 (85)	96,640 (2400)	413,036 (15,473)	11.02 (0.17)	597 (15)

*M*_n_ = number average of molecular weight (MW), *M*_w_ = weight-average MW, *M*_z_ = z average MW, PDI (polydispersity index) = *M*_w_/*M*_n._

**Table 6:** SEC results of spruce wood hemicelluloses (g·mol^−1^, SD are in the parentheses).

Sample	*M*_n_	*M*_w_	*M*_z_	PDI
REF	6941 (115)	45,278 (210)	143,947 (5873)	6.52 (0.14)
160-TW	6256 (721)	44,305 (3052)	141,816 (1549)	7.10 (0.33)
180-TW	6869 (32)	27,205 (176)	85,926 (2590)	3.96 (0.01)
210-TW	5300 (371)	13,597 (21)	27,680 (1906)	2.57 (0.18)
160-TW-XE	7066 (213)	42,191 (207)	122,628 (9765)	5.97 (0.15)
180-TW-XE	7186 (88)	24,929 (2)	60,378 (752)	3.47 (0.04)
210-TW-XE	4930 (30)	13,274 (347)	35,063 (3818)	2.69 (0.09)

**Table 7:** Differences in the FTIR absorbance intensities of spruce wood cellulose.

Wavenumber (cm^−1^)	Δ_TW_ (%)			Δ_TW-XE_ (%)		
	160 °C	180 °C	210 °C	160 °C	180 °C	210 °C
898	17.49	10.06	−11.39	4.46	−9.85	−1.60
1030	19.20	9.37	3.22	−3.90	−4.35	6.67
1053	10.33	3.96	−2.35	−5.58	−6.43	2.28
1103	−3.03	−1.66	−6.02	−6.31	−8.83	−3.39
1160	−7.09	−6.08	−14.63	−11.82	−13.91	−13.57
1202	−12.91	−10.24	−7.75	−10.80	−18.16	−12.38
1261	2.36	24.42	68.46	−1.36	25.89	50.82
1315	−2.24	−0.09	−19.33	1.88	−9.09	−18.81
1334	−2.87	−2.80	−20.37	1.82	−8.60	−16.86
1429	−8.40	−6.49	6.10	−6.15	−10.17	3.15
1450	−0.54	6.64	27.21	3.34	1.08	25.32
1512	−42.82	74.72	1107.14	40.74	122.37	923.57
1644	11.18	21.35	78.89	−4.81	12.99	67.16
1727	26.01	58.44	221.10	14.94	85.31	193.35
2895	1.62	−0.05	−2.28	3.66	−2.83	6.53
3338	1.18	−4.80	−3.67	2.73	0.13	7.01

## Data Availability

The original contributions presented in the study are included in the article; further inquiries can be directed at the corresponding author.
